# Domestic and Family Violence Affecting Children and Young People from Culturally and Racially Marginalized Migrant Backgrounds in Australia: A Scoping Review of Child Experiences and Service Responses

**DOI:** 10.1177/15248380241265386

**Published:** 2024-07-27

**Authors:** Gemma Tarpey-Brown, Jess Kirwan, Sarah Wise, Eva Alisic, Cathy Vaughan, Karen Block

**Affiliations:** 1 University of Melbourne, Carlton, VIC, Australia

**Keywords:** cultural contexts, domestic violence, children exposed to domestic violence, disclosure of domestic violence

## Abstract

In Australia, children and families from culturally and racially marginalized (CARM) migrant backgrounds experience a range of compounding structural and interpersonal factors that limit help-seeking and exacerbate the impacts of domestic and family violence (DFV). This scoping review examines the current state of knowledge on how children and young people from CARM migrant backgrounds experience DFV, and the services that respond to DFV including child protection services. A systematic search was conducted across PsycINFO, MEDLINE, and CINAHL databases and Google Scholar, alongside a complementary grey literature search. Articles were included in the review if participants were from CARM migrant backgrounds, and the article included information related to children and young people’s experiences of DFV, and the DFV service system. The review found 19 articles that met selection criteria. Due to limited research on this topic in Australia, most articles focused on children and young people’s experiences shared through parental, carer or service provider perspectives. To our knowledge, this is the first scoping review to examine how children and young people from CARM migrant backgrounds experience DFV. Findings demonstrate children and young people are victim-survivors of multiple forms of DFV. Children and young people’s engagement with the DFV service system is often accompanied by feelings of fear and distrust. Findings suggest that to strengthen system responses to DFV, services must build their capability to implement intersectional approaches that simultaneously support the safety and well-being of both the child and the non-violent parent or carer.

## Introduction

Despite over half the Australian population being born overseas or having a parent born overseas (ABS, 2022) the prevalence of domestic and family violence (DFV) perpetrated against children and young people from culturally and racially marginalized (CARM) migrant backgrounds remains relatively unknown (Abdul Rahim et al., 2023). DFV refers to any physical, sexual, emotional, psychological, or economic abuse, including behaviors that coerce, control or dominate, and that occur in current or former family or intimate relationships (*Family Violence Protection Act* 2008, 2023; Hegarty et al., 2000). DFV is a gendered phenomenon predominantly used by men against women and children (ABS, 2023; Heise et al., 2002; Nixon & Humphreys, 2010); however, it is only recently that children and young people have started to be seen as victim-survivors of DFV in their own right.

Prevalence rates of DFV perpetrated against adult women from CARM migrant backgrounds in high-income countries vary; however, emerging evidence indicates this population may experience DFV at higher rates than the non-migrant population (Rees et al., 2022; Vaughan et al., 2016; Webster et al., 2019). Families from CARM migrant backgrounds experience a range of compounding structural and interpersonal factors that limit help-seeking and exacerbate the impacts of DFV (Block et al., 2022; Hourani et al., 2021; Segrave, 2018; Vasil, 2023; Vaughan et al., 2016). In order to mitigate compounding factors and ultimately increase the safety of children and young people, it is critical we build evidence of how children and young people from CARM migrant backgrounds experience DFV. It is also necessary to understand how the DFV service system responds to the needs of CARM migrant background children and young people. We recognize that child protection services are a major component of the service system response to DFV when children are involved. In this review, we will discuss a range of service responses and highlight the key role of child protection services in supporting children from CARM migrant backgrounds who have experienced DFV.

The population of interest includes people from first- and second-generation migrant backgrounds, the latter referring to individuals born in Australia to migrant parents. The term CARM is used to specify groups who may be culturally and/or racially marginalized due to perceived differences from the white Anglo-settler majority in Australia (Diversity Council of Australia, 2023).^1^ We define children as people under the age of 18 and acknowledge them as competent social actors with the right to participate (Åkerlund & Gottzén, 2017; Convention on the Rights of the Child, 1989). We define young people in this review as people aged between 16 and 18 years of age (United Nations, 2024).

### Current Service System Responses to DFV for Children and Young People from CARM Migrant Backgrounds

Service system responses to DFV that enhance the safety and well-being of families require collaboration across a range of different organizations including specialist DFV, legal, child protection, education, health, family, and perpetrator support services. Different organizations across the service system may understand DFV through a sector-specific lens influencing the ways in which DFV is conceptualized and therefore responded to (Macvean et al., 2018). These sector-specific conceptualizations of DFV are relevant in interactions between specialist DFV services, child protection services, and families. Mothers experiencing DFV are often fearful of or constrained in their engagement with child protection services as the system places responsibility to protect children on the mother. Supported by a “failure to protect” approach, child protection services have been found to victim-blame mothers rather than empower them to safely stay with their children (Azzopardi, 2022; Humphreys & Absler, 2011; Meyer, 2015). In opposition to this, historically DFV response services have viewed the mother as the main victim-survivor and the child as a secondary or indirect victim, rarely incorporating children’s needs into assessment and response frameworks. Following recent large-scale reforms to the DFV service system in some Australian jurisdictions, children’s needs are beginning to be assessed (through mothers) and integrated into assessment plans. For families from CARM migrant backgrounds, systemic racism within the service system complicates current responses to DFV, creating an environment of fear and mistrust (Berkman et al., 2022; Bourke et al., 2019; Maturi, 2023). Such fear and mistrust have been recognized in previous research with women from CARM migrant backgrounds and has led to the identification of a critical nexus between reluctance to report DFV and the fear of child removal by child protection services (Block et al., 2022; Ibrahim, 2022; Kaur & Atkin, 2018; Vaughan et al., 2016, 2020). Developing a nuanced understanding of this nexus is a key priority if we are to comprehensively recognize, respond to and prevent DFV.

To better identify how this nexus operates, it is necessary to briefly examine Australian child maltreatment legislation. Child maltreatment legislation varies across state jurisdictions. Only four states and territories^2^ specifically refer to DFV as a reason a child needs statutory protection and may subsequently be placed in out of home care (OOHC). Other states and territories do not specifically mention DFV and instead refer to psychological and emotional harm that may impact a child’s emotional and intellectual development (Australian Institute of Family Studies, 2021). Despite these legislative inconsistencies across jurisdictions, fear of child removal in CARM migrant communities following a formal disclosure of DFV is not unfounded, given the racialized history of child protection services in Australia (Oates, 2020; O’Donnell et al., 2019; Read, 2020). Aboriginal and Torres Strait Islander children have been forcibly separated from their families, communities and country since the beginning of British colonization in Australia (Yoorrook Justice Commission, 2023) and continue to be grossly overrepresented in Australian child protection systems (Davis, 2019; Morgan et al., 2023). Although the experiences of Aboriginal and Torres Strait Islander children are outside the scope of this review, their histories and experiences of forced child removal are an important reference point when examining how CARM migrant families may experience DFV services as racialized systems of care.

Our scoping review has two aims: to examine the current state of knowledge on how children and young people from CARM migrant backgrounds experience DFV; and to examine existing literature on how these communities experience the services that respond to DFV. Below, we outline our theoretical framework informed by an intersectional ethics of care, before moving on to present our methods and review findings.

## Theoretical Framework

We use a critical understanding of intersectional care ethics to frame this review. Intersectional care ethics recognizes care as a relational practice embedded within power relations operating along axes of race, gender, class, and culture (Hankivsky, 2014). An intersectional ethics of care requires practitioners to explicitly recognize harmful power relations (Clark-Kazak, 2023) and therefore has the capacity to enhance social justice frameworks implemented across DFV and social welfare services (Juujärvi et al., 2020; Pease et al., 2017). For Hankivsky (2014) and Tronto (2013, 2020), an intersectional ethics of care demonstrates how dominant power relations control and shape who is cared for, and by whom. Due to global systems of oppression, it is often those most in need of care who receive the least. This notion is supported by Raghuram (2019), who argues racial hierarchies underpin our understandings of who gets to be cared for, and what practices are defined as appropriate forms of care.

In Australia, British colonial models of care shape legislative and cultural understandings of what constitutes appropriate care (Ramsay, 2016). Such understandings of care may fail to recognize the intricate ways racialized hierarchies of knowledge and power shape what practices are deemed appropriate forms of care. As mothers from CARM migrant backgrounds come into contact with the DFV service system, their care practices are viewed through a racialized colonial model that may misinterpret or misunderstand forms of care as harmful for the child. The family may then be monitored in a way that views the mother as negligent, or as failing to protect her child (Maturi, 2023; Rogerson, 2012). In such circumstances, the service system does not recognize the mother’s own status as a victim-survivor of DFV who also needs support and potential protection. By using intersectional ethics of care theory to frame this review, we aim to highlight the multiple forms of power and control operating across structural, systemic, and interpersonal levels that simultaneously affect the safety of the mother and child, and emphasize the need to enhance service system responses that holistically support mother and child victim-survivors together.

## Methods

We conducted the review between March 2023 and March 2024 in line with the Preferred Reporting Items for Systematic Reviews and Meta-Analyses (PRISMA) extension for scoping reviews (Tricco et al., 2018). The review consisted of four phases: the development of a scoping review protocol (Peters et al., 2022); identifying and selecting relevant studies; extracting and charting data; and summarizing and reporting the results. Critical reflexivity was an integral component of the review process (Arksey & O’Malley, 2005) as it was essential we recognized our own positionality as Anglo-Celtic white-settlers living and working on unceded Aboriginal and Torres Strait Islander land. We acknowledge that child protection systems in Australia continue to have resonance and historical continuity with settler-colonial practices of structural violence (Davis, 2019; Referendum Council, 2017).

### Search Strategy

We conducted the search across PsycINFO, MEDLINE, CINAHL databases, and Google Scholar (first 10 pages). Search terms were developed with guidance from an academic librarian using Boolean search strategies to combine three key concepts (Box 1). A complementary systematic search of gray literature was conducted using similar search terms. We also screened the reference lists of included articles for additional sources.

**Box 1.** Search Terms Used in Three Scientific and Health Related Databases (PsycINFO, MEDLINE, CINAHL) and Google Scholar.

*Concept 1* (child* or teen* or youth or young person or young people or adolescen*).mp. Concept 2 (refugee* or migrant* or immigrant or asylum seeker*).mp. Concept 3 family violence.mp. or exp Domestic Violence or IPV.mp. or intimate partner violence.mp. or interpersonal violen*.mp. or (maltreat* or abuse or assault*).mp. Place australia*.mp.

### Selection Criteria

Studies included were peer-reviewed articles, book chapters, books, and publicly available gray literature. There were no methodological limitations on sources. Research must have been at least partially conducted in Australia due to the specificities of legislation relating to DFV and studies must have been published between 2003 and 2023. Studies were included if participants were from CARM first- or second-generation migrant backgrounds. Participants were children and young people, or primary carers of children, and young people who spoke about their children’s experiences of DFV. We also included studies where participants were service providers who worked closely with the target group and provided information on how children and young people experienced DFV and moved through the service response system. Studies were excluded if there was no specific information about how children and young people under 18 had experienced DFV and/or interacted with the DFV service system. There were no restrictions placed on visa status or period of time since settling in Australia. Unpublished studies were excluded from the review.

### Data Extraction and Analysis

**Figure 1.**
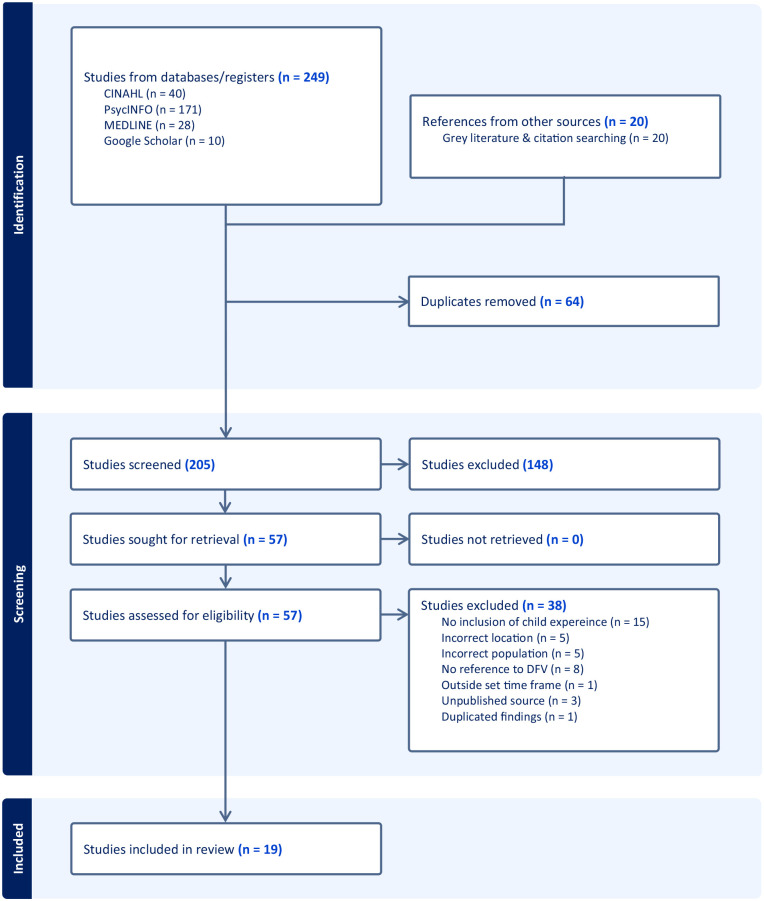
PRISMA flow diagram of the study selection process.

Our search returned a total of 269 articles. We imported all references into Covidence and removed 64 duplicates. Titles and abstracts were screened independently by the first and second authors (*n* = 205). We conducted full-text reviews in line with eligibility criteria (*n* = 57) and 19 articles met selection criteria (Figure 1). Disagreements on selection were resolved by discussion, and if consensus was not reached, the article was referred to all members of the research team. We developed a data extraction form in Covidence including type of evidence source; publication and audience; location; methodology; population and sampling; aim; presence of child voice^3^; intersectionality; key findings; and limitations. To enhance the rigor of the review the first and second authors extracted data independently then collaboratively synthesized and reached consensus on all extracted information (Arksey & O’Malley, 2005). We combined the data extraction forms into a single chart (Table 1) and conducted a descriptive thematic analysis which we present below.

**Table 1.** Children and Young People from CARM Migrant Backgrounds Experiences of DFV—Study Characteristics, Aims, and Relevant Findings.

Author	Aim	Methodology	Population	Relevant Findings
Abdul Rahim et al. (2023)	To describe the cumulative incidence of child protection system contact, maltreatment type, source of reports to age 7 years for culturally and linguistically diverse (CALD) Australian children across South Australia.	Quantitative	Children aged between 0 and 7 born in South Australia (SA) who had their birth registered in SA and were enrolled in public schools between 2009 and 2015 (*n* = 76,563).	Provided incidence data relating to CALD families’ contact with the child protection system in SA. Findings demonstrated CALD children aged between 0 and 7 years in South Australia had lower risk of contact with the child protection system from the point of notification to Out of Home Care (OOHC). It is the first Australian study to examine the risk of child protection contact for CALD children at population level.
Basu and Isaacs (2019)	To describe the cultural profile of transcultural patients presenting to a Child and Adolescent Mental Health Service (CAMHS).	Quantitative	Children and young people 18 years and under from a “transcultural” background who attended the service between 2013 and 2017 (*n* = 101).	Provided an overview of the most common social and psychosocial stressors experienced by children and young people from diverse cultural backgrounds with mental ill-health in regional Australia. The study highlighted connections between presentation at Child and Adolescent Mental Health Services and adverse childhood experiences including domestic violence, “family conflict,” and other forms of child maltreatment.
Burke and Paxman (2008)	To identify demographic characteristics, placement history, current placement characteristics, types of services provided and the well-being of a sample of children and young people from Arabic-speaking and Vietnamese backgrounds in OOHC.	Mixed methods	Arabic-speaking and Vietnamese children in OOHC (*n* = 28).	Provided analysis of cultural appropriate practices used by child protection services (CPS) when supporting children from non-English speaking backgrounds placed in OOHC. Discussed systemic barriers preventing culturally appropriate practices. Recommendations were to increase cultural diversity of carer recruitment; multicultural OOHC caseworkers; culturally specific foster care projects; culturally specific resources for carers and clients; and correct translation of key documents for carers and parents.
de Anstiss (2023)	To determine the nature and scope of help-seeking behavior for young women from refugee backgrounds and examine how specific socioecological factors may influence help-seeking.	Qualitative	Cisgender young women aged 18–28 years who had experienced partner violence in a heterosexual relationship between the ages of 13 and 19 years and were living in South Australia (*n* = 17), and women “helping professionals” (*n* = 5).	Intimate partner violence (IPV) prevention, response and support services utilized a framework for adult victim-survivors. There were limited formal supports specializing in dedicated services for young victim-survivors, and even fewer support services responding to the needs of young people from refugee backgrounds who experienced a range of systemic barriers to accessing help. Findings demonstrated fear of child removal was substantiated by CPS responses. In instances of child removal, whether temporary or permanent, information provided to young mothers was inadequate and participants reported no support or referral before, during or after their children had been taken from their care.
Fisher (2013)	To investigate the interrelationship between experiences of domestic violence, and changed and changing gender and family roles and responsibilities in five African refugee background communities.	Qualitative	Two population groups: people from Somalian, Sierre Leonean, Ethiopian, Liberian and Sudanaese Communities (*n* = 54); and professional who provide support to those communities (*n* = 24)	African community members did not view physical violence toward children as a form of domestic violence. Rather, it was seen as a necessary form of discipline performed by the parent to help guide the child. However, parents noted that since arriving in Australia, this form of physical violence was not acceptable. Community members identified that the service response to domestic violence in Australia as “pro-women and children” which is to men’s detriment.
Fitz-Gibbon et al. (2023)	To elevate the voices of young people with lived experience of family violence (FV), and who have navigated the service system in Victoria.	Qualitative	Children and young people aged between 10 and 25 with lived experience of FV (*n* = 17). Over 1 third of participants were from migrant and refugee backgrounds (*n* = 5).	Provided insight into how children and young people experience FV, how they conceptualize and maintain their own and their siblings’ well-being when living with violence, and their interactions with the FV response system. The study highlighted the importance of system enhancements, ensuring the availability of child-centric spaces, age-appropriate supports and individualized responses, safe and trauma informed practices, and greater consistency in how mandatory reporting requirements are discussed with young people.
Hatoss (2022)	To explore perspectives of South Sudanese parents on parenting conflicts in Australia and to increase understanding of the sources of cross-cultural and intergenerational conflicts within families.	Qualitative	South Sudanese parents of refugee background living in Australia who had arrived between 2005 and 2008. Participants were between 24 and 48 years of age. The sample included seventeen mothers and five fathers (*n* = 22).	Children viewed physical punishment from parents as a form of violence and self-reported to CPS that their parents were abusing them. The article also provided parental perspectives on the use of physical violence against children as a form of punishment and behavior management. CPS were seen by parents to encroach on “inside” family dynamics and rules, imposing Australian cultural values.
Kaur (2012)	To review available Australian research on the interface between Child Protection and families from CALD and refugee backgrounds to identify gaps within research knowledge and propose future research priorities.	Literature review	Not applicable	Highlighted the significant gaps in practice knowledge and research on how CALD and refugee children and families engage with system responses to child abuse and neglect. The review found 13 publications between 1996 and 2012. Studies identified in the review found DFV was one of the most common issues experienced by CALD and refugee background families notified into the CPS. DFV did not occur as an isolated form of harm, most often co-occurring with a range of other factors. Key recommendations were developed at policy, service and practitioner level.
Kaur (2019)	To examine child maltreatment within ethnic minority communities and outline the complex interactions of race, ethnicity, social class, patriarchy, religion, immigration status, and linguistic diversity to demonstrate how these impact ethnic minority families in contact with CPS	Qualitative	Families from ethnic minority backgrounds who had come into contact with CPS.	Presented a cross-cultural child protection assessment framework. The framework was developed to support practitioners working in CPS and included consideration of ethnic and cultural factors; diverse child rearing practices; settlement experience; and consideration of refugee and asylum seeker experience prior to arrival in Australia.
Lewig et al. (2009)	To examine why recently arrived families from refugee backgrounds are presenting to the child protection system and to identify culturally appropriate strategies and models for intervention.	Mixed methods	Two population groups: child protection workers in South Australia; and families referred to CPS who were recent humanitarian arrivals (*n* = 81)	Provided data relating to children from refugee backgrounds contact with the child protection system in South Australia. Children and families experienced a range of intersecting factors that resulted in notification to CPS including DFV, substance misuse, limited understanding of complex service system, social isolation, limited English language proficiency, mental ill health, and pre-migration experiences of trauma and torture.
Losoncz (2016)	To examine how CPS can more effectively engage and intervene with South Sudanese families.	Qualitative	Two population groups: recently arrived South Sudanese parents; and community and youth workers	Explored perspectives of South Sudanese parents who experienced conflict and violence within the family. Examined how newly arrived South Sudanese parents engage with child protection and other violence response and social services, including schools, police and youth organizations. Parents saw these services as intervening and supporting their children to leave which further challenged parental power and agency. This contributed to distrust of formal services.
Maturi (2023)	To explore how front line domestic violence workers who support women from refugee and migrant backgrounds understand the concept of empowerment.	Qualitative	Frontline domestic violence workers from refugee and non-refugee backgrounds (*n* = 31)	Critically investigated how the concept of empowerment has come to be understood as an individual process related to self-agency. Formal response services were said to actively disempower children and mothers from refugee and migrant backgrounds who experienced gendered violence, through coercion. Such coercion referred to CPS strongly encouraging women to leave their relationships, despite the fact that for some women staying in the relationship may be the safest option for them and their children due to structural inequalities. Child protection also used surveillance and monitoring methods that traumatizing of both child and adult survivors of domestic violence.
Posselt et al. (2015)	To identify risk factors and patterns of comorbidity development in young people from refugee backgrounds living in a disadvantaged urban region of Adelaide, South Australia	Qualitative	Two population groups: young refugee and migrant people aged between 15 and 25 years (*n* = 15); and service providers (*n* = 15).	Did not utilize a DFV lens, however it did describe instances of DFV. Physical and verbal abuse were referred to as intergenerational conflict, with 86% of young people from refugee backgrounds reporting they had experienced some form of this. The repercussions of DFV within the home are manifold and may contribute to comorbidity of alcohol and other drugs and mental health disorders.
Ramsay (2016)	To explore how the child welfare system in Australia is a basis of governmentality, that not only operates to protect children but simultaneously functions as an instrument to govern mothers to fit into an idealized standard of Australian citizenship.	Qualitative	Women aged between 18 and 40 who had arrived in Australia as refugees following extended periods of time in refugee camps (*n* = 35). Most were mothers.	Provided a critical analysis of the racialized history of the child welfare system in Australia, and how this system is today experienced by African women from refugee backgrounds who are victim-survivors of FV. The article highlighted a number of intersecting social factors that may prevent children who are forcibly removed by CPS from being able to return to their birth parents. Some of these factors were compounded by experiences of forced migration. The article argues racialized social institutions and the child welfare system does not provide culturally safe care for children or their families.
Rees et al. (2022)	To explore the prevalence and risk factors of IPV among Australian-born and refugee-background women.	Mixed methods	Two population groups: women from refugee backgrounds from conflict affected countries with Arabic as a national language as well as Sudan and Sri Lanka (*n* = 685); and women non-refugee backgrounds (*n* = 650) who attended public antenatal clinics in Sydney and Melbourne.	The first prospective cohort study in Australia to measure IPV alongside co-occurring mental health disorders among mothers from refugee backgrounds, compared to women born in Australia. Women from refugee backgrounds experienced significantly higher rates of IPV pre- and postpartum compared to the Australian born control group.
Renzaho et al. (2011)	To document parenting styles among African migrants living in Melbourne and assess how intergenerational issues related to parenting in a new culture impact on family functioning and the modification of lifestyles.	Qualitative	Individuals born in Ethiopia, Sudan and Somalia, aged 13+ who had been in Australia between 0 and 15 years (*n* = 85).	Investigated issues related to parenting in a new culture. Did not explicitly explore FV, however provided examples of how physical violence toward children is used as a form of discipline. Findings show corporeal punishment including smacking and starvation are used by parents against their children. These forms of violence are supported by parents’ understanding of what is expected and acceptable.
Sawrikar (2019)	To explore what service providers need to meet the needs of ethnic minority children and families who have come to the attention of child protection authorities and have substantiated reports of domestic violence.	Mixed methods	Two population groups: CALD family members aged between 22 and 67 years (*n* = 29); and CPS caseworkers aged between 23 and 59 years (*n* = 17).	Proposed there are three inter-related issues that affect how children and families from ethnic minorities interact with the domestic violence (DV) service system: the need to protect the family name; to promote family cohesion; and experiencing racism. Historically, the issue of DV and child maltreatment in ethnic minority communities had been understood through a cultural lens that perpetuated the notion violence may be linked to cultural factors and practices. DV is a generalist risk factor of child maltreatment in all populations; however, families from ethnic minorities have diverse needs.
Stratford et al. (2022)	To better understand the views and experiences of newly arrived young people and services that work with them in relation to FV and accessing support.	Qualitative	Two population groups: young women aged between 18 and 25 years from diverse cultural backgrounds (*n* = 10); and service providers who worked with newly arrived young people (*n* = 26).	Newly arrived young people experienced diverse and overlapping forms of FV. Young people expressed frustration with current responses to FV, stating prevention interventions are inadequate and that their experiences with response services have been negative due to racism and other factors. Young people identified extensive barriers to access support for FV. FV was not always perpetrated by a partner, with young people providing examples of multi-perpetrator abuse from a father, brother—or someone outside of the immediate family. Key recommendations for service system reform and prevention system reform were provided.
Zannettino (2012)	To examine why recently arrived families from refugee backgrounds are presenting to the child protection system and to identify culturally appropriate strategies and models for intervention.	Mixed methods	Three population groups: families engaged with the child protection system who had come from countries where Australia had been receiving recent humanitarian entrants (*n* = 81 families); child protection practitioners (*n* = 55); and community members from Africa, the Middle East and Vietnam.	For Liberian refugee women and children, using physical violence as a form of child discipline was viewed as a cultural phenomenon which may lead to the general acceptability of other forms of FV in the home. Fear of family breakdown and forced separation from their partners were barriers to reporting FV and engaging with FV services.

## Findings

First, we provide an overview of the studies included in the review. We then present our thematic analysis of key findings which produced four themes: *How diverse forms of violence affect children and young people; Trajectories of care through CPS; How fear shapes children and young people’s engagement with social services*; and *The influence of structural conditions and social factors on experiences of DFV*. These findings are framed by an intersectional ethics of care recognizing care as a deeply relational practice (Clark-Kazak, 2023). This framework emphasizes both children and their caregivers are victim-survivors who need shared protection and care in the context of DFV (Nissen & Engen, 2021).

### Description of Studies and the Absence of Children’s Voices

In line with inclusion criteria all articles were from Australia (*n* = 19) with the majority including research conducted in South Australia (*n* = 5) and Victoria (*n* = 4). Evidence types included primary research articles (*n* = 13), research reports from government and non-government sources (*n* = 4), a literature review (*n* = 1), and a book chapter (*n* = 1). Most articles used qualitative research methodologies (*n* = 12) with semi-structured interviews the main method of data collection (*n* = 9). Over a third of the articles (*n* = 9) used multiple methods of data collection including case study analyses, consultations, focus group discussions and surveys. The most common theoretical framework was intersectionality (*n* = 5), followed by socio-ecological theory (*n* = 4) and feminist theory (*n* = 3). Over a third of articles (*n* = 8) did not state if a theoretical framework was used to guide the analysis. Of the 19 articles in the review, only four drew from research conducted directly with children and young people who had experienced DFV. All other empirical research articles involved parents, carers, and service providers.

### How Diverse Forms of Violence Affect Children and Young People

All articles in the review described multiple forms of DFV perpetrated against children and young people. DFV was most often perpetrated by a male family member (de Anstiss, 2023; Fisher, 2013; Hatoss, 2022; Kaur, 2012; Lewig et al., 2009; Ramsay, 2016; Sawrikar, 2019; Stratford et al., 2022; Zannettino, 2012). Multi-perpetrator violence from members of extended family, siblings, and community leaders was described in two studies (Lewig et al., 2009; Stratford et al., 2022). Physical violence was the most common form of DFV reported (Basu & Isaacs, 2019; de Anstiss, 2023; Fisher, 2013; Hatoss, 2022; Kaur, 2019; Lewig et al., 2009; Losoncz, 2016; Posselt et al., 2015; Renzaho et al., 2011; Stratford et al., 2022; Zannettino, 2012).

There was notable variation in how articles defined DFV. Kaur (2019) identified five specific forms of child abuse which included witnessing DFV (other forms were physical; neglect; sexual; and emotional). Behaviors constituting DFV were not always defined as such when perpetrated against children, as some articles classified DFV against children as a form of emotional abuse (Abdul Rahim et al., 2023; Basu & Isaacs, 2019; Sawrikar, 2019). Specific forms of coercive control were described, including partners threatening to remove children from their mothers, psychological and financial abuse (de Anstiss, 2023; Stratford et al., 2022). Other forms of DFV included deliberate deprivation of food and starvation (Renzaho et al., 2011; Stratford et al., 2022); neglect (Abdul Rahim et al., 2023; Kaur, 2019; Lewig et al., 2009); spiritual abuse (Stratford et al., 2022); child slavery-like practices (Stratford et al., 2022); forced child marriage (Kaur, 2019); technology facilitated abuse (Stratford et al., 2022); and distinct forms of migration-related abuse including the threat of deportation (Stratford et al., 2022).

Intimate partner violence (IPV) perpetrated against young people was discussed in two studies (de Anstiss, 2023; Stratford et al., 2022). Stratford et al. (2022) conducted focus groups with newly arrived young women from refugee backgrounds. Participants described experiencing multiple forms of IPV including physical violence, financial abuse, and technology-facilitated abuse which involved the misuse of photos in online environments. de Anstiss (2023) interviewed young women from refugee backgrounds who had experienced IPV between the ages of 13 to 19. Participants discussed their lived experiences and previous understandings of IPV. When experiencing IPV at such a young age, they did not recognize the violence as a problem until it reached crisis point. Some participants went on to describe a hierarchy of violence in which physical assault was not deemed serious unless it resulted in hospitalization. Due to the severity of physical IPV, half of the adolescent mothers in the study had been hospitalized. One adolescent girl experienced such severe physical violence that she gave birth prematurely. In instances of psychological and emotional abuse, most participants stated they did not understand these behaviors as forms of violence until after they had left the relationship.

### Trajectories of Care Through CPS

Engagement with CPS was a key component of the service system response to DFV involving children. Studies indicated contradictory findings where children from CARM migrant backgrounds were both over and under-represented in state systems of care (Abdul Rahim et al., 2023; Burke & Paxman, 2008). The only population prevalence study included in this review used a linked data set in South Australia to describe the cumulative incidence of child maltreatment between 0 and 7 years of age, type of maltreatment and source of notification for CARM migrant compared to non-CARM migrant background children (Abdul Rahim et al., 2023). Findings indicated that by age 7, culturally and linguistically diverse (CALD) children were at lower risk of all forms of contact with the child protection system than non-CARM migrant children. The authors noted that, given the lack of an official definition for CALD children, identifying how and at what rate this group interacted with the child protection system was challenging.

#### Initial Engagement Pathways and Mandatory Reporting

Mandatory reporting is the legal requirement for certain professional groups to report disclosure, or reasonable belief, of child abuse to child protection authorities. As noted, mandatory reporting legislation differs across state jurisdictions in Australia. In the state of Victoria where this review was conducted, mandatory reporters include teachers, early childhood practitioners, people in religious ministry, police officers, midwives, nurses, and other registered health practitioners (Department of Families, Fairness and Housing, 2023). Fitz-Gibbon et al. (2023) found practitioners did not make it clear to children and young people when they are legally mandated to make a report to child protection if they believe a child needs protection. Most reports to child protection for children from CARM migrant backgrounds came from police and teachers (Abdul Rahim et al., 2023; Lewig et al., 2009).

Some studies noted that for young people, knowledge of mandatory reporting acted as a barrier to help-seeking (de Anstiss, 2023; Stratford et al., 2022). Pregnant adolescent girls reported that knowing health practitioners were mandated to report abuse was a barrier to accessing pre-natal care. They also stated they avoided medical attention after episodes of DFV for fear of being reported to child protection (de Anstiss, 2023). In contrast, Hatoss (2022) described instances of children self-reporting and requesting to be removed from their parents’ care, which was a source of concern for some CARM migrant parents.

#### How Experiences of Child Removal Substantiate Fears

The forced removal of children was a pressing concern for parents and communities (de Anstiss, 2023; Hatoss, 2022; Lewig et al., 2009; Ramsay, 2016). CARM migrant communities feared CPS due to the threat of surveillance and potential child removal (de Anstiss, 2023; Maturi, 2023; Ramsay, 2016). de Anstiss (2023) reported that girls experiencing IPV chose not to report or seek formal help due to fear of child protection removing their children. This fear was exacerbated for some women survivors of IPV who had experienced war-related trauma prior to arriving in Australia, due to interactions with violent and corrupt services in their countries of origin whereby their husbands were taken from their homes by police for extended periods of time and for unexplained reasons (Zannettino, 2012). Such experiences meant that once in Australia, women continued to fear statutory services.

Mothers’ fear of child removal were substantiated in de Antiss’ qualitative study (2023) with young women who had experienced IPV between 13 and 19 years of age. All adolescent mothers who reported IPV (*n* = 6) had their children removed. In three cases, the removal was permanent, and no young mothers received any professional support from services before, during or after intervention from CPS. In case studies outlined by Ramsay (2016) children were forcibly removed from their mothers’ care due to reports of DFV perpetrated by mothers’ partners. In findings from interviews with service providers in Queensland, Maturi (2023) found that the level of surveillance and monitoring from child protection was so intense that a mother and her children became traumatized. This led to the mother consistently making decisions about her life based on how child protection might respond, despite her child’s case being closed.

#### Beyond Notification of Abuse to Engagement with Social Support Services

There was limited information on the trajectories of care for children from CARM migrant backgrounds following a report to child protection, and/or once removed from parental care. Studies investigating children’s experiences of child protection after a report of maltreatment had been substantiated identified several systemic barriers and challenges impacting positive outcomes for children and their families. Burke and Paxman (2008) examined a sample of child protection case files (*n* = 28) of children from Arabic and Vietnamese-speaking backgrounds who had been placed in OOHC in New South Wales due to reasons including DFV. Case workers demonstrated inconsistent approaches when placing children from CARM migrant backgrounds in an OOHC arrangements, whereby cultural matching with the foster carer was not always a consideration.

CPS were considered to hold culturally specific understandings of care shaped by racialized assessment frameworks that may include biases related to a family’s racial, class, cultural, gendered, or linguistic identity (Kaur, 2019; Ramsay, 2016; Sawrikar, 2019). Ramsay (2016) provided an example in her ethnographic study where an African refugee woman named Camille was experiencing DFV and had her children forcibly removed by CPS. Camille was only able to see her children through supervised visits held at child protection offices in a room filled with toys and games. Camille did not consider that her parenting ability was being assessed based on her engagement with these toys and play-based activities. However, child protection workers informed her she had been assessed as demonstrating a lack of attachment to her children, given, she did not engage in play with them when in the visitation room. In this instance, Camille’s parenting ability was measured in line with normative western cultural understandings of good care giving practices.

### How Fear Shapes Children and Young People’s Engagement with Social Services

Beyond CPS, children and young people interacted with and at times sought support from teachers (Posselt et al., 2015; Stratford et al., 2022), youth workers (Losoncz, 2016; Stratford et al., 2022), social workers (de Anstiss, 2023), police (Lewig et al., 2009; Losoncz, 2016; Ramsay, 2016), crisis accommodation (Stratford et al., 2022), family violence workers (Fitz-Gibbon et al., 2023), and multicultural specialist services (Kaur, 2012). Interactions with these services was often in informal settings, or short-term, as many did not provide child-centered approaches to care. Children and young people struggled to access support services due to their limited awareness of what services were available. Services also had restrictive age limits preventing young people under the age of 18 from accessing supports (de Anstiss, 2023; Fitz-Gibbon et al., 2023; Stratford et al., 2022). Limited engagement with formal services was present from a young age, with infants from CARM migrant backgrounds found to be significantly less likely than children with Australian-born mothers to be engaged with postnatal care programs (Rees et al., 2022).

Children and young people perceived services as culturally unsafe—especially for newly arrived migrants—unaffordable, and gender inappropriate (Posselt et al., 2015; Stratford et al., 2022). Children and young people emphasized services using binary language (e.g., victim and perpetrator) perpetuated considerable misunderstandings of the dynamics of DFV (Fitz-Gibbon et al., 2023). Accessing services in rural and regional locations was seen to increase risk of isolation and limit access to education and anonymity. For adolescents experiencing IPV, keeping their relationship secret from families was a distinct barrier to seeking support which further increased their vulnerability to sexual coercion and blackmail (de Anstiss, 2023). Other fears held by children and young people were wide ranging and included fear of migration-related consequences such as deportation (Stratford et al., 2022); fear of retribution (Stratford et al., 2022); fear of causing a family breakdown (de Anstiss, 2023; Zannettino, 2012); fear of violence from community for bringing outside attention to DFV (Stratford et al., 2022); fear of services which stemmed from knowledge of other young people’s negative experiences (Fitz-Gibbon et al., 2023; Losoncz, 2016; Posselt et al., 2015; Stratford et al., 2022); and fear of disrupting established care arrangements (Stratford et al., 2022).

### The Influence of Structural Conditions and Social Factors on Experiences of DFV

Children and young people’s experiences of DFV were compounded by a range of structural conditions and social factors that affected how they accessed support services. Interpersonal and systemic racism were the dominant forms of discrimination experienced by children and young people engaged with the DFV service system (Basu & Isaacs, 2019; Lewig et al., 2009; Posselt et al., 2015; Ramsay, 2016; Sawrikar, 2019). At the structural level, migration policies dictated children and young people’s eligibility for certain support services. Two studies outlined how migration status was weaponized by perpetrators, especially regarding threats of deportation and child abduction (Losoncz, 2016; Stratford et al., 2022).

Social factors related to migration contributed to how parents understood and performed caring duties. There was prominent discussion of how shifting power dynamics within CARM migrant families during resettlement led to a change in familial roles and expectations, which in turn contributed to generational differences in understandings of care and abuse (Basu & Isaacs, 2019; Fisher, 2013; Hatoss, 2022; Losoncz, 2016; Posselt et al., 2015). Newly arrived young people described grappling with internal conflict between their increasing understanding of DFV as defined by Australian law, and attitudes and behaviors of families and communities (Basu & Isaacs, 2019; Losoncz, 2016; Posselt et al., 2015; Renzaho et al., 2011; Stratford et al., 2022). For some communities, the notion of a “cultural clash” was discussed in relation to forms of disciplinary practices being categorized as abuse under Australian law (Fisher, 2013; Hatoss, 2022; Zannettino, 2012). This led communities to feel disempowered and distrustful of support services as they were concerned children would report them for maltreatment. Mothers also stated that their experiences of war-related trauma affected their ability to effectively care for their children, resulting in perceptions of neglect by services (Zannettino, 2012).

Families from CARM migrant backgrounds experienced multiple intersecting social concerns which co-occurred with the perpetration of DFV. These co-occurring factors compounded children and young people’s experiences and resulted in significant social, health and economic impacts. Studies did not frequently specify who in the family was experiencing the co-occurring issue. Information was limited on how these issues may have specifically compounded or exacerbated children’s experiences of DFV or have been weaponized by the perpetrator. Substance misuse, mental ill-health, and poverty were the most common factors occurring in families who sought support for DFV or who were notified to child protection (Basu & Isaacs, 2019; Burke & Paxman, 2008; de Anstiss, 2023; Kaur, 2019; Lewig et al., 2009; Maturi, 2023; Posselt et al., 2015; Ramsay, 2016; Renzaho et al., 2011). Other factors included social isolation (de Anstiss, 2023; Losoncz, 2016; Maturi, 2023; Ramsay, 2016); homelessness (Kaur, 2019; Maturi, 2023; Posselt et al., 2015); incarceration of a parent (Burke & Paxman, 2008); court proceedings (Lewig et al., 2009; Losoncz, 2016); physical illness (Lewig et al., 2009); developmental concerns (Rees et al., 2022); and difficulties associated with English language proficiency (Lewig et al., 2009; Maturi, 2023). These social, health, and economic factors demonstrated the need for multi-sectoral collaboration across the service response system informed by understandings of intersectionality. Such collaboration may work to ensure care is provided to children and their families in a way that minimizes harm while recognizing that structural and systemic conditions shape how mothers and children from CARM migrant backgrounds experience DFV.

## Discussion

In Australia, the current state of knowledge regarding how children and young people from CARM migrant backgrounds experience DFV and subsequent service responses is narrow. Findings indicate children and young people experienced diverse forms of DFV and should be recognized as victim-survivors in their own right, rather than as “witnesses” to DFV (Callaghan et al., 2018; Katz et al., 2020; Lamb et al., 2018). These findings also apply to children and young people under the age of 18 who experienced IPV (de Anstiss, 2023; Stratford et al., 2022). Only four studies included the voices and perspectives of children and young people with lived experience of DFV. Other studies focused on either parents’ perspectives of how children experienced DFV, or the perspectives of service providers working with children and young people (Table 2).

**Table 2.** Summary of Critical Findings.

Children and young people from CARM migrant backgrounds are affected by multiple forms of DFV, including coercive control, and migration-specific forms of violence.
There was a paucity of research conducted with children and young people with lived experience of DFV. Most research was conducted with adult victim survivors, carers, and service providers.
Within the DFV service system children and young people were at times considered indirect victims of DFV, with services using language that stated the child “witnessed DFV” or was “exposed to DFV.” This detracted from children and young people’s lived experiences of being victim-survivors.
Families from CARM migrant backgrounds who were engaged in the DFV service system had multiple co-occurring social factors that compounded their experiences of DFV. This affected access to and engagement with services and effective support.
Across jurisdictions the prevalence of children and young people from CARM migrant backgrounds experiencing DFV was not known. The proportion of this population engaged with child protection and other social services that respond to DFV was also unknown.

*Note.* CARM = culturally and racially marginalized; DFV = domestic and family violence.

Studies highlighted inconsistent definitions of what constitutes violence perpetrated against children and young people. Some jurisdictions had a separate DFV category, whereas others classified DFV as a form of “emotional abuse” and none defined coercive control against children. Such inconsistencies limit accurate prevalence reporting, as well as understanding the risks and impacts of DFV experienced by children. Inconsistencies in reporting abuse type may also contribute to understandings of children and young people as “appendages” to their mothers, given that “witnessing DFV” was noted as a form of abuse in some studies (Kaur, 2019; Lewig et al., 2009; Sawrikar, 2019).

Findings also show the needs of both the mother and child were rarely considered simultaneously. Child protection responses summarized in this review did not consistently consider the mother’s need for care. Rather, the care, protection, and safety needs of a child or mother tended to be considered separately and assessed by different services (Macvean et al., 2018). A siloed service response may make it challenging to develop a comprehensive understanding of the patterns of violence used by the perpetrator, especially in situations where coercive control is used. This suggests that current responses to children and young people from CARM migrant backgrounds who experience DFV may be enhanced by using an intersectional ethics of care (Hankivsky, 2014; Raghuram, 2019). A response framed by an intersectional ethics of care may ensure both mother and child are provided holistic care and protection. Such care simultaneously recognizes intersecting social, cultural, gendered, racial, and class-based factors that may shape how families experience DFV, and subsequently seek help and support.

Despite Australia’s highly diverse population (ABS, 2022), the review found minimal prevalence data on DFV experienced by children and young people. Most studies in the review were qualitative and conducted with small participant samples, often drawn from specific ethnic communities. While qualitative studies provide rich granular data, the small number of quantitative studies indicate there is significant scope for further research into the population prevalence of children and young people from CARM background who experience DFV. One study gathered population prevalence data on children from CARM migrant backgrounds who had been reported to child protection; however, the study did not investigate the proportion of notifications linked to DFV (Abdul Rahim et al., 2023). Other studies found CARM migrant communities to be disproportionately underrepresented in systems of care, supporting the argument this population is invisible across DFV and social services systems (Ghafournia & Easteal, 2018).

Findings of our review examining the nexus between fear of child removal and disclosing DFV indicated this nexus was present at individual and community levels (Kaur & Atkin, 2018). At the individual level, mothers’ fears of having their children removed by CPS after formally disclosing DFV were substantiated (de Anstiss, 2023; Maturi, 2023; Ramsay, 2016). In most cases, children were temporarily removed from their mothers. However, permanent child removal was more common in cases where the mother was an adolescent (de Anstiss, 2023). Maturi (2023) highlighted how, for one mother, her fear of further involvement from CPS was so intense that it severely impacted her parenting and decision-making processes while recovering from DFV.

These fears were exacerbated by social services in the provision of racialized and gendered assessments (de Anstiss, 2023; Fitz-Gibbon et al., 2023; Kaur, 2019; Ramsay, 2016; Sawrikar, 2019). The issue of racialized, or culturally unsafe, assessments was raised by Kaur (2019) who discussed the occurrence of “false positive” assessments, where a caseworker incorrectly assumes a cultural act or practice is abusive toward a child. This monocultural or westernized understanding of care may deem mothers from CARM migrant backgrounds as incapable of providing appropriate care, especially in situations where DFV is present. This meant that mothers were effectively held responsible for the violent actions of perpetrators. Placing responsibility on the mother, who is a victim-survivor of DFV alongside her children, rather than on the perpetrator minimizes the perpetrator’s accountability in the violence. This demonstrates responses to DFV found in the studies operated in direct opposition to an intersectional ethics of care (Juujärvi et al., 2020; Pease et al., 2017). Instead, assessments were used to justify the forced removal of children from their mother’s care, substantiating fears of statutory CPS and disregarding evidence on how crucial the mother–child relationship is in shaping children’s experiences of DFV (Hester, 2011; Swanston et al., 2014).

## Limitations

The dearth of relevant evidence which includes children’s voices and perspectives meant our review incorporated studies that did not directly include children and young people as participants. This resulted in children’s experiences being presented by a third party, most often parents, carers, or service providers, and meant that risk and protective factors of children and young people were not sufficiently captured in this review. A further limitation of the review relates to identification. Children and young people from CARM migrant backgrounds are identified using a diverse range of factors including country of birth, migration status, ethnicity, and language status. There is no singular way to define this cohort. Most studies included in this review used different identifying factors to define children and young people from CARM migrant backgrounds. This means that children with certain migration experiences, such as those on temporary visas seeking asylum or who were detained in Australian immigration detention centers, may be underrepresented in this review.

## Implications for Policy, Practice, and Research

A key step in preventing DFV is generating evidence to understand how children and young people from CARM migrant backgrounds experience DFV. Listening to and directly involving child participants must be a central part of research conducted to inform the development of policy and practice in DFV prevention (Table 3) (de Anstiss, 2023; Fitz-Gibbon et al., 2023; Renzaho et al., 2011; Stratford et al., 2022). This will enhance understanding of protective factors and limit the use of deficit-based approaches when providing care and support to children moving through the DFV response system. The review highlighted that by introducing continuity in the reporting of families’ CARM migrant status across jurisdictions may strengthen the DFV response system by ensuring services correctly identify children’s cultural, linguistic, religious, and other needs.

**Table 3.** Summary of Implications for Practice, Policy, and Research.

Practice	• Workers in the DFV service system should complete training on utilizing intersectional theory and frameworks to increase their capability to recognize intersecting structural and systemic discrimination that compound experiences of DFV. • Implementation of mandatory cultural safety training that includes identification and discussion of key visa types, and how these visa types may affect children and young people’s access to specific social, health, and community services. • Training for child protection workers emphasizing the importance of a holistic approach that considers the safety of the child and non-violent parent simultaneously and together.
Policy	• Establish a clear definition of “CARM migrant background” that is used across state jurisdictions when collecting demographic information from children, young people, and families entering the DFV service system. • Recognize children and young people who experience DFV as victim survivors in their own right across relevant state and national policy, rather than referring to children as “witnesses” or being “exposed” to violence.
Research	• Develop research methodology identifying the prevalence of children and young people from CARM migrant backgrounds who have been notified to child protection, and who have experienced DFV. • Conduct research with children and young people from CARM migrant backgrounds who have lived experience of DFV to ensure their voices are heard and amplified. • Ensure strengths-based research questions are incorporated into studies to assist in identifying protective factors and strategies children use to maintain their own safety and well-being when experiencing, or at risk of, DFV.

*Note.* CARM = culturally and racially marginalized; DFV = domestic and family violence.

It is critical service providers conceptualize children and young people as direct victim-survivors of DFV with their own distinct protective factors and lived experience, rather than as “witnesses” of violence. This conceptualization must occur while maintaining the non-violent parent is also a victim-survivor. By using a framework informed by an intersectional ethics of care, the children’s needs, and the need of the non-violent parent can be considered simultaneously and their relationship supported alongside the dynamics, risks and impacts of DFV. In doing so, services may be able to recognize how processes of structural oppression and DFV compound to create distrust and fear among CARM migrant background families, and begin to work with families in a more holistic way. This can be enhanced by services and practitioners using an intersectional approach when working with children and young people from CARM migrant backgrounds. An intersectional understanding of DFV allows services to identify and respond to multiple co-occurring social factors and structural conditions which influence how children and young people experience DFV, and seek help and support.

## Conclusion

The paucity of research examining how children and young people from CARM migrant backgrounds experience and are affected by DFV supports the concern that these communities are mostly invisible within the DFV and child protection service system. This is despite women and children from CARM migrant backgrounds experiencing a range co-occurring structural and social factors which may amplify the impacts of DFV and create substantial barriers to accessing culturally safe and supportive services. Findings also indicate a need to explore how the pervasive sense of fear, which prevents CARM migrant women and children from voluntarily engaging with DFV services, might be mitigated through responses informed by an intersectional ethics of care. Research that works directly alongside this cohort of children and young people is integral to the development of child centered and holistic DFV response and prevention frameworks which must be embedded into existing service systems.

## Notes

1. We use the term CARM as it explicitly names marginalization based on culture and race as a shared experience for black, brown, Asian, and other non-white people in Australia. The term CARM was developed by the Diversity Council of Australia, and it problematizes the framing of white Anglo Celtic as the “norm,” whereby people from other cultures and racial backgrounds are frequently othered by being referred to as “diverse.”

2. Northern Territory, Tasmania, New South Wales, and Western Australia (Australian Institute of Family Studies, 2021).

3. Child voice was defined as the direct involvement of children and young people under the age of 18 as participants in the study.
